# Unraveling the Diagnosis of Hypocomplementemic Urticarial Vasculitis Syndrome

**DOI:** 10.7759/cureus.57723

**Published:** 2024-04-06

**Authors:** Syed Bukhari, Mohamed Ghoweba, Syed Khan, Geoffrey Ouma

**Affiliations:** 1 Vascular Medicine, Cleveland Clinic, Cleveland, USA

**Keywords:** literature review of disease, rare skin disease, inflammatory arthritis, eosinophilia, leukocytoclastic vasculitis (lcv), hypocomplementemic urticarial vasculitis

## Abstract

Hypocomplementemic urticarial vasculitis syndrome (HUVS) is a rare condition characterized by immune complex-mediated urticarial lesions with histological features of leukocytoclastic vasculitis, low serum complement levels, and is frequently associated with systemic manifestations. Its pathophysiology is poorly understood. We present a patient who presented with abdominal pain and skin rash. Extensive work-up was performed including skin biopsy, and the presence of angioedema, oral ulcers, low complement level, leukocytic vasculitis, and persistent eosinophilia ultimately led to the diagnosis of HUVS. This case highlights the importance of recognizing and differentiating HUVS from other cutaneous diseases, which in turn helps to optimally manage these patients.

## Introduction

Hypocomplementemic urticarial vasculitis syndrome (HUVS), also known as McDuffie syndrome, is a rare and distinct type of urticarial vasculitis with multiorgan involvement whose exact pathophysiology remains unknown. HUVS is often accompanied by autoimmune diseases, particularly systemic lupus erythematosus (SLE), and ~ 50% of HUVS patients are diagnosed with SLE. Diagnosis of HUVS relies on the Schwarz criteria which includes clinical and laboratory findings. Two major criteria (chronic recurrent urticaria for more than six months and hypocomplementemia) and at least two minor criteria (leukocytoclastic vasculitis on skin biopsy, arthralgias and arthritis, glomerulonephritis, uveitis or episcleritis, abdominal pain, and positive C1q antibody test) are needed for a diagnosis of HUVS. The possible mechanisms of vascular damage in HUVS include immune complex-mediated, T-lymphocyte response, and anti-C1q antibody. We present a case of a patient with a known history of rheumatoid arthritis, who presented with abdominal pain and oral ulcers, and extensive workup led to the diagnosis of HUVS.

## Case presentation

**Figure 1:**
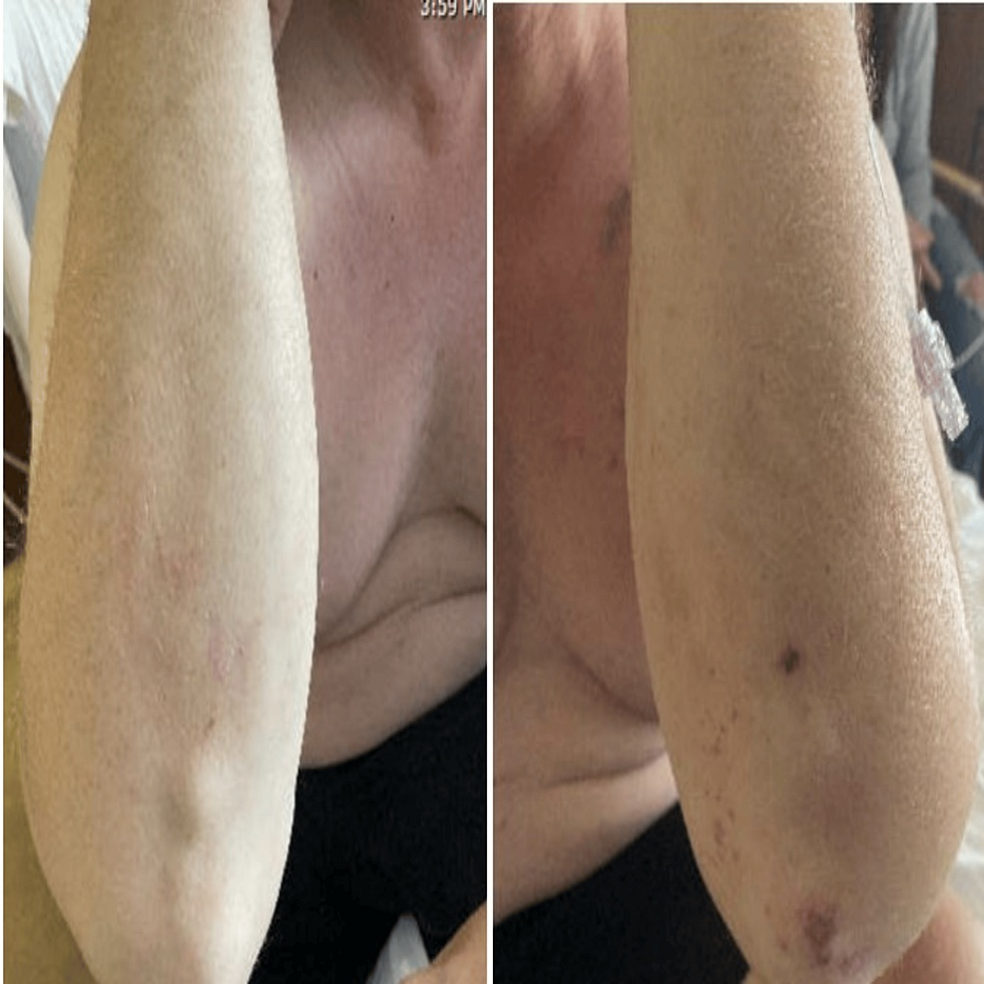
Purpuric rash on left elbow, and urticarial rash on the right proximal forearm

**Figure 2:**
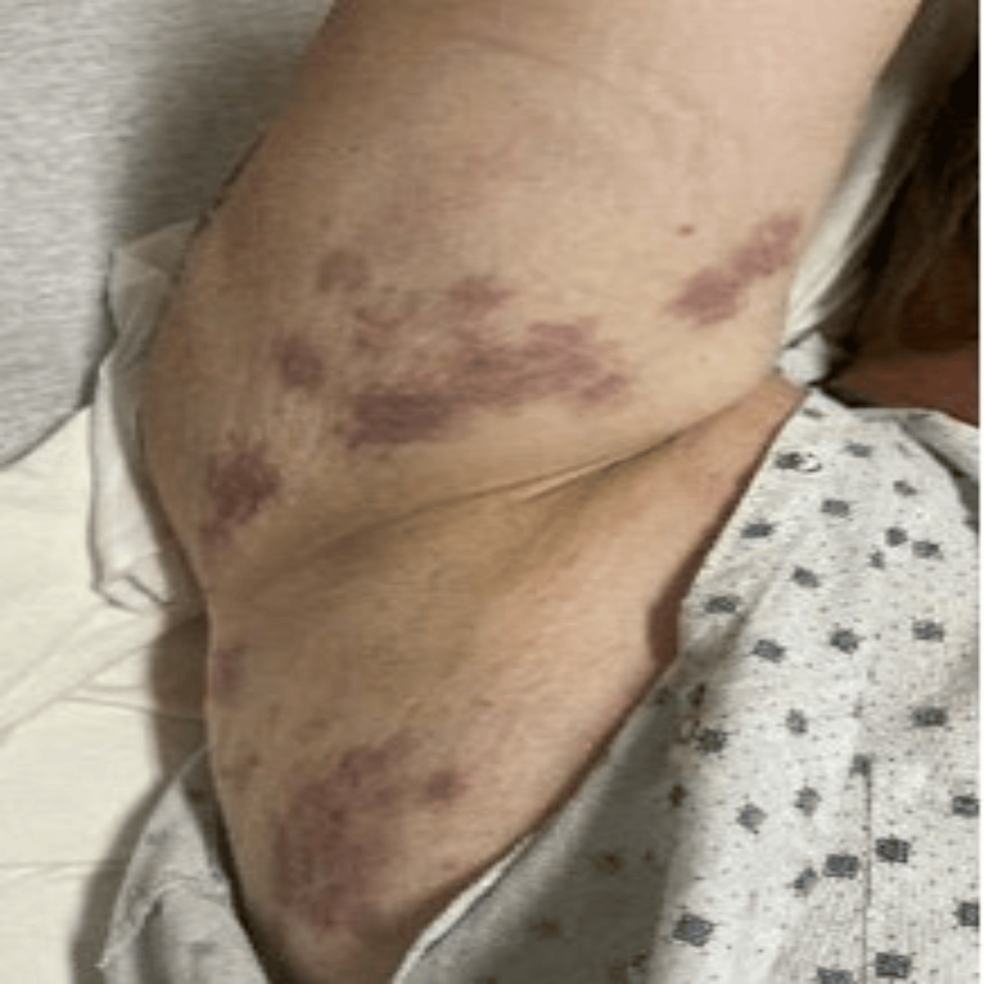
Purpuric rash in the axillary area

**Figure 3:**
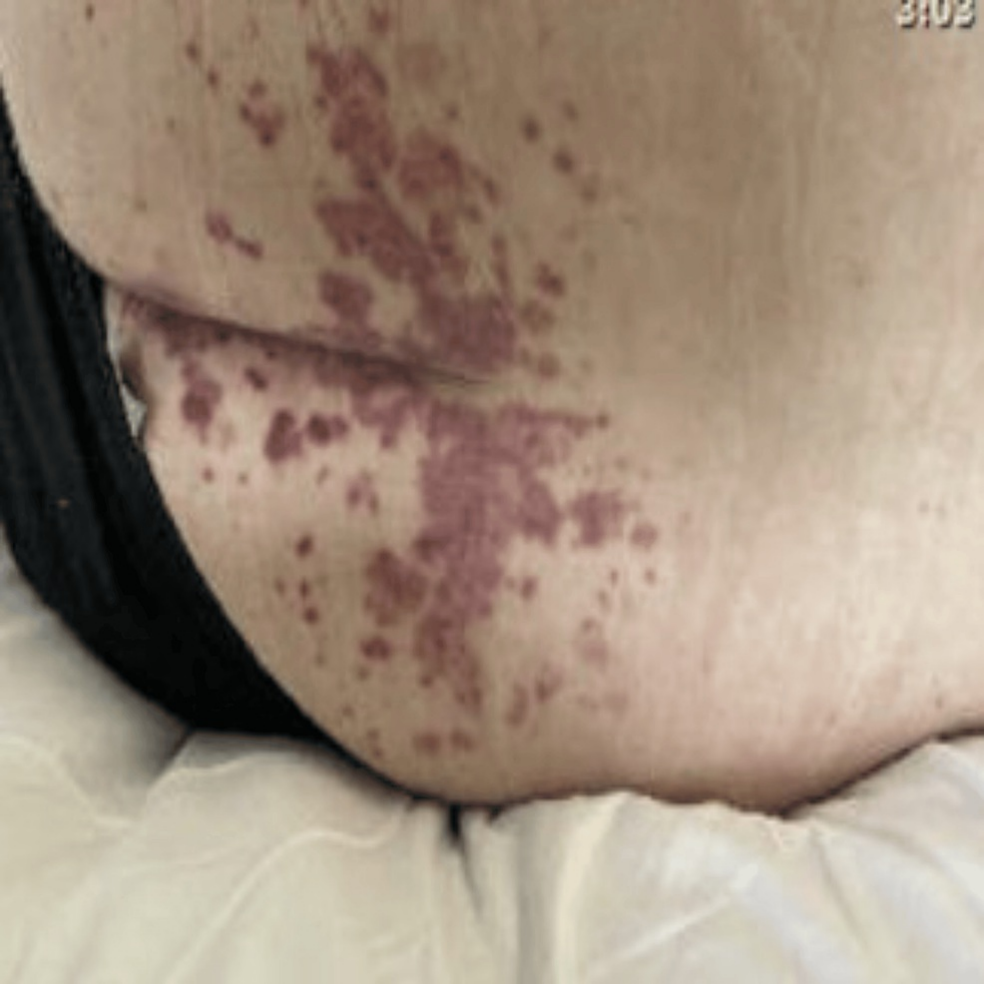
Purpuric rash in the buttocks

A 44-year-old male presented to the emergency department with abdominal pain and chronic recurrent urticarial skin rash for nine months. His past medical history was significant for seropositive rheumatoid arthritis, and venous thromboembolism for which he was anticoagulated with apixaban. On arrival, his blood pressure was 135/78 mmHg, pulse of 75 beats per minute, and saturating 99% on room air. Physical examination showed oral mucosal ulcerations and diffuse purpuric and urticarial lesions on multiple locations in the body (Figures 1-3). 

He underwent computed tomography that showed extensive mural thickening throughout the ileum, ascending colon, hepatic flexure, and duodenum suggestive of intestinal angioedema. His laboratory workup showed eosinophilia, and initial inflammatory markers revealed elevated C-reactive protein and erythrocyte sedimentation rate (Table 1).

**Table 1:** Pertinent laboratory test results

Test	Result	Reference range
C-reactive protein	4.7 mg/dL	<0.9 mg/dL
Erythrocyte sedimentation rate	122 mg/dL	<0.9 mg/dL
ANA	1:80 (homogenous)	<1:40
DNA antibodies	<12.3 IU/ml	<30 IU/ml
Smith antibodies	<0.2 U	<0.1 U
Ribonucleoprotein antibodies	<0.2 U	<0.1 U
Rheumatoid factor	232 IU/ml	0-20 IU/ml
C3 complement	79 mg/dL	86-166 mg/dL
C4 complement	11 mg/dL	13-46 mg/dL
C1q complement	<50 ug/ml	109-242 ug/ml
Anti-C1q antibody, IgG	43 U	0-19 U
Human immunodeficiency virus	Negative	Negative
C1 esterase inhibitor	92%	> 42%
JAK2 V617F mutation	Negative	Negative

Elevated inflammatory markers, purpuric lesions, and a history of inflammatory disease prompted further rheumatological workup. His inflammatory/rheumatological evaluation was largely unrevealing, notably negative anti-double stranded and anti-smith antibodies, low complement levels C3 and C4, and negative c-ANCA, p-ANCA, human immunodeficiency virus, and cryoglobulin. During hospitalization, eosinophilia persisted, and workup from an infectious and hematologic standpoint including bone marrow biopsy was unremarkable for parasitic infection or primary hematologic malignancy. The patient also underwent a biopsy of the skin rash that came back positive for leukocytoclastic vasculitis. Due to the presence of leukocytoclastic vasculitis, inflammatory rheumatoid arthritis, hypocomplementemia, oral ulcers, persistent eosinophilia, and angioedema, rheumatology and hematology services strongly suspected HUVS. Finally, the anti-C1q antibody was ordered which came back positive, establishing the diagnosis of HUVS.

He was started on methylprednisone 60 mg daily with improvement in his symptoms. He was also initiated on famotidine and cetirizine given the urticarial picture of his rashes. In addition to steroids, dapsone was added to his regimen with folic acid. Finally, hydroxychloroquine was added at the time of discharge. On a two-month follow-up, his rash was resolved.

## Discussion

Differential diagnoses for this case included other causes of urticaria including aquagenic, androgenic, pressure-induced, cold-induced, and cholinergic causes. Specifically, the patient’s presentation had to be differentiated from chronic spontaneous urticaria, bullous pemphigoid, Henoch-Schönlein purpura, cryoglobulinemic vasculitis, rheumatoid vasculitis, eosinophilic cellulitis, cutaneous mastocytosis, neutrophilic urticarial dermatosis, Behcet syndrome and erythema multiforme [1]. Given the patient’s clinical presentation meeting the criteria stated below and histopathological findings, a diagnosis of HUVS syndrome was established.

HUVS is a rare condition characterized by immune complex-mediated urticarial lesions with histological features of leukocytoclastic vasculitis, and low serum complement levels, and is frequently associated with systemic manifestations. It has a reported incidence of 0.5/100,000 persons and usually presents in the 30s to 40s age group with a female: male ratio of 8:1 [2,3].

The pathophysiological processes underlying HUVS remain poorly understood. These are hypothesized to involve an immune complex-mediated mechanism (Type III hypersensitivity reaction). Anti-C1q antibodies bind to the collagen domains of the C1q resulting in the formation of immune complexes that deposit on the vasculature. This leads to complement activation (C3a and C5a) via the classical pathway which results in damage of the endothelium [3]. IL-1 has been postulated to be involved as well. This theory is further bolstered by the successful treatment of HVUS with IL-1 inhibitors such as anakinra and the monoclonal antibody against IL-1β canakinumab [1].

Patients with HUVS typically present with recurrent and chronic urticarial rash. If present, systemic manifestations commonly include musculoskeletal symptoms in the form of arthralgia or arthritis; ocular with conjunctivitis, episcleritis, uveitis; gastrointestinal with abdominal pain, nausea, vomiting, diarrhea; pulmonary with asthma or chronic obstructive pulmonary disease; renal involvement ranging from mild nonprogressive disease to rapidly progressive renal disease resulting in end-stage renal disease [1]. Renal involvement occurs in up to 50% of patients with HUVS [4].

Diagnosis of HUVS relies on the Schwarz criteria which includes clinical and laboratory findings. Two major criteria (chronic recurrent urticaria for more than six months and hypocomplementemia) and at least two minor criteria (leukocytoclastic vasculitis on skin biopsy, arthralgias and arthritis, glomerulonephritis, uveitis or episcleritis, abdominal pain, and positive C1q antibody test) are needed for a diagnosis of HUVS [5]. HUVS can occur alone or in association with other auto-immune diseases in 25% of patients including systemic lupus erythematosus, Sjogren syndrome, rheumatoid arthritis, juvenile rheumatoid arthritis, and mixed connective tissue disorder. The diagnostic criteria for HUVS frequently overlap with those of SLE. Notably, it has been reported that 54% of HUVS patients are eventually diagnosed with SLE during the follow-up period [6]. The presence of ANA with a titer of >= 1:80 effectively rules out HUVS and warrants a diagnosis of SLE [4].

There is no consensus regarding the treatment for HUVS, hence, management is tailored based on each patient’s clinical picture. For patients in which serum complement levels remain normal during an attack, HUVS is usually self-limiting. Symptomatic control with antihistamines and nonsteroidal anti-inflammatory agents may be helpful. Patients might benefit from commonly prescribed medications for SLE such as low-dose prednisone, hydroxychloroquine, or dapsone. Severe systemic involvement often requires more aggressive therapies such as immunosuppressive agents with cytotoxic agents including cyclophosphamide, cyclosporine A, azathioprine, mycophenolate mofetil, and methotrexate, either alone or in combination with high-dose steroid therapy with prednisone. Despite its transient effect, plasmapheresis has been utilized to reduce the levels of circulating immune complexes [3,5].

## Conclusions

Hypocomplementemic urticarial vasculitis syndrome is a rare immune complex-mediated condition characterized by urticarial/purpuric lesions with systemic manifestations, low serum complement levels, and histological features of leukocytoclastic vasculitis. Its pathophysiology is poorly understood. It often occurs in patients with underlying inflammatory diseases, like systemic lupus erythematosus or rheumatoid arthritis. The diagnosis is based on clinical presentation, laboratory workup showing hypocomplementemia, and histological confirmation of leukocytoclastic vasculitis. Treatment is symptomatic with prednisone, hydroxychloroquine, or dapsone, along with antihistamines in most cases.
